# Recanalization Treatment of Acute Ischemic Stroke Caused by Large-Artery Occlusion in the Elderly: A Comparative Analysis of “the Elderly” and “the Very Elderly”

**DOI:** 10.1155/2021/3579074

**Published:** 2021-10-06

**Authors:** Qi Wang, Yi-Qun Zhang, Han-Cheng Qiu, Yin-Dan Yao, Ao-Fei Liu, Chen Li, Wei-Jian Jiang

**Affiliations:** ^1^The PLA Rocket Force Characteristic Medical Center, The Teaching Hospital of Soochow University, Beijing, China; ^2^Department of Vascular Neurosurgery, New Era Stroke Care and Research Institute, Department of Neurology, Beijing Rehabilitation Hospital, Capital Medical University, Beijing, China; ^3^Department of Vascular Neurosurgery, New Era Stroke Care and Research Institute, The PLA Rocket Force Characteristic Medical Center, Beijing, China

## Abstract

**Objective:**

To assess whether the effectiveness and safety of recanalization therapy for acute ischemic stroke (AIS) caused by large-artery occlusion (LAO) differ between patients aged 60–79 years and patients aged ≥80 years.

**Methods:**

We analyzed prospective data of patients with LAO (≥60 years) who underwent recanalization therapy at the Department of Vascular Neurosurgery, New Era Stroke Care and Research Institute, PLA Rocket Force Characteristic Medical Center, from November 2013 to July 2017. The data were compared between elderly patients (60–79 years) and very elderly patients (≥80 years). The effectiveness of recanalization therapy was evaluated using the 90-day modified Rankin scale (mRS) score, while safety was assessed by the rates of symptomatic intracranial hemorrhage (SICH) and mortality within 30 days.

**Results:**

A total of 151 patients with AIS induced by LAO were included in this study. Seventy-three patients (48.3% [73/151]) had an overall favorable outcome (mRS score 0–2) after treatment. A higher proportion of patients in the elderly group showed a favorable outcome compared with the very elderly group (58.6% [34/58] vs. 41.6% [39/93], respectively; *P* = 0.046). The incidence of SICH (12.7% vs. 16.13%, respectively; *P* = 0.561) and mortality (10.3% vs. 7.5%, respectively; *P* = 0.548) within 30 days was not significantly different between the two groups.

**Conclusion:**

Recanalization treatment of LAO is more effective in elderly patients compared with very elderly patients, while the safety of recanalization treatment is comparable between these two groups.

## 1. Introduction

Acute large-artery occlusion (LAO) has become the most important cause of acute ischemic stroke (AIS) worldwide and is related to the high recurrence rate of ischemic stroke and poor outcomes [1]. Endovascular thrombectomy (ET) and intravenous alteplase thrombolysis (IVT) are currently the preferred vascular recanalization treatments for AIS.

Stroke morbidity and mortality increase with age, and the absolute number of patients with fatal stroke is likely to increase steadily due to population aging, a continued increase in traditional risk factors, and poor management [2].

Age is usually regarded as an important factor affecting the choice of treatment and prognosis, and this consideration is particularly prominent in individuals aged over 80 years [3–6]. However, few randomized controlled trials have investigated large-vessel recanalization treatment in patients aged over 80 years.

With the development of recanalization treatment technology and population aging, the treatment of acute LAO in patients over 80 years of age is receiving increasing attention. In 2013, the Chinese Stroke Association and the American Heart Association/American Stroke Association modified AIS recombinant tissue plasminogen activator (rt-PA) IVT guidelines. Specifically, an age of greater than 80 years was no longer an absolute exclusion criterion; instead, it became a relative exclusion criterion (level II evidence and grade B recommendation) [7, 8]. Moreover, the inclusion criteria for AIS recanalization therapy in clinical practice have been further expanded. Very elderly patients with low National Institutes of Health Stroke Scale (NIHSS) scores, an Alberta Stroke Program Early Computed Tomography (ASPECT) score of <6, and posterior circulation infarction have been gradually indicated to undergo revascularization therapy.

At the same time, an increasing number of single-center trials began to include very elderly patients over 80 years of age into the scope of intravascular recanalization treatment and compared them with elderly patients aged less than 80 years [9]. It has been emphasized that the safety of ET is comparable between patients aged over 80 years and those aged less than 80 years [10]. However, the relationship between recanalization therapy and clinical outcomes in very elderly patients (those aged over 80 years of age) is not fully understood. Therefore, this study was aimed at assessing whether the benefits and safety of recanalization therapy for acute LAO and AIS are comparable between patients ≥ 80 years of age (very elderly patients) and patients aged 60–79 years (elderly patients) to provide a basis for clinical treatment. Thus, we aimed to assess whether there is a difference in the effectiveness and safety of recanalization therapy for AIS caused by LAO between elderly patients and very elderly patients. We also aimed to provide a basis for the future selection of patients suitable for acute stroke recanalization treatment and to investigate whether recanalization treatment has a better effect in elderly patients compared with very elderly patients. The findings of this study may inform future treatment selection.

## 2. Materials and Methods

### 2.1. Patient Information

This study was a retrospective study that included patients aged ≥60 years with AIS who were admitted continuously to the Department of Vascular Neurosurgery, New Era Stroke Care and Research Institute, PLA Rocket Force Characteristic Medical Center, from November 2013 to July 2017. Patients were divided into the elderly group (60–79 years) and the very elderly group (≥80 years). Patients' baseline characteristics, imaging results, and treatment plans were recorded. This study was approved by the Ethics Committee of PLA Rocket Force Characteristic Medical Center, and written informed consent was obtained from all patients.

The inclusion criteria were as follows: (1) aged ≥60 years; (2) met the AIS diagnostic criteria, including clear neurological deficit, presence of an infarct on diffusion-weighted imaging, and no cerebral hemorrhage on head computed tomography (CT) [11]; (3) presence of LAO at the internal carotid artery (ICA), vertebral artery, basilar artery (BA), or main middle cerebral artery (M1), which was related to the stroke event and confirmed by magnetic resonance angiography or digital subtraction angiography; and (4) an onset time window of within 4.5 hours for IVT and within 6 hours for intravascular treatment in patients with anterior-circulation disease and within 24 hours for patients with posterior-circulation disease.

The exclusion criteria were as follows: (1) coagulation dysfunction, mental disorders, or malignant tumors; (2) liver or kidney dysfunction, organ diseases, or an incomplete medical history; (3) acute cerebral hemorrhage, systemic infection, or blood system disease; (4) bilateral or multiple large-artery disease, perforating artery disease or small-artery disease, a history of surgical or interventional treatment for aortic valve stenosis, or other diseases affecting clinical follow-up and judgment; (5) refusal or failure to comply with the treatment plan or failure to complete follow-up; and (6) participation in other clinical trials.

### 2.2. Treatment Plan

#### 2.2.1. IVT

For patients who met the requirements for IVT and who had no contraindications to IVT, 0.9 mg/kg rt-PA (Boehringer Ingelheim Pharmaceutical Co., Ltd., Germany) was administered. For patients with a higher bleeding risk, the doctor exercised the decision to administer 0.6 mg/kg rt-PA based on experience.

#### 2.2.2. ET

All surgeries were performed by an experienced neurointerventionist (Professor Jiang Weijian). General or local anesthesia was used depending on the patient's condition and tolerance. Thrombus location was determined using angiography, and the Solitaire AB stent removal device was preferentially used for thrombus removal. The degree of recanalization was measured by modified thrombolysis in cerebral infarction (mTICI), with grade ≥ 2b representing successful recanalization and grade 3 representing complete recanalization [12].

#### 2.2.3. Blood Pressure Management

To prevent ischemia–reperfusion injury after recanalization, blood pressure was kept below 180/105 mmHg before surgery and maintained at around 80% of the baseline level but not less than 90/60 mmHg after vascular recanalization.

#### 2.2.4. Antiplatelet Drugs

After ET, patients were routinely treated with dual antiplatelet therapy (oral aspirin 100 mg/day and clopidogrel 75 mg/day). Three months later, single-agent antiplatelet therapy with aspirin 100 mg/day or clopidogrel 75 mg/day was administered long term.

#### 2.2.5. Statins

Patients diagnosed with cerebral infarction were immediately administered statin treatment. For patients with severe atherosclerosis or emergency stent implantation, intensive statin treatment was used.

### 2.3. Outcome Assessment

The mRS score 90 days after treatment was used as the evaluation standard to determine recovery of neurological function. An mRS score of 0–2 was defined as a good functional prognosis, and an mRS score of 3–6 was defined as a poor prognosis [13]. The mRS score was recorded by a neurologist who followed up patients by telephone or during clinic visits.

The incidence of SICH and mortality within 30 days after treatment was recorded. SICH was defined as subarachnoid hemorrhage or cerebral parenchymal hemorrhage with neurological deficit.

### 2.4. Statistical Analysis

SPSS 22.0 statistical software was used for data analysis. Quantitative data are expressed as mean ± standard deviation or median (interquartile range (IQR)) depending on whether the data were normally distributed. The *t*-test or the rank-sum test was used to identify differences between two individual groups. Count data are expressed as the number of cases or as a percentage and were compared using the chi-square test. A repeated-measures analysis of variance was used to compare NIHSS and mRS scores at different time points in each group. A *P* value of <0.05 was considered statistically significant. Variables with *P* values of <0.1 in a univariate analysis were included in a multivariate regression analysis. Confounding factors were controlled by stratified analysis and propensity score matching.

## 3. Results

### 3.1. Patients' Baseline Characteristics

**Figure 1:**
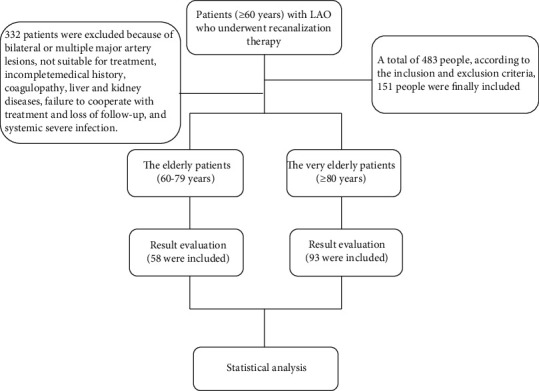
Research flow chart.

A total of 483 patients were enrolled from November 2013 to July 2017, according to the inclusion and exclusion criteria; 151 people were finally included, all of whom were diagnosed with AIS caused by LAO (Figure 1). There were 74 men (49% [74/151]) and 77 women (51% [77/151]). Among them, 38.4% of patients (58/151) belonged to the elderly group (60–79 years of age), and 61.6% of patients (93/151) belonged to the very elderly group (80–93 years of age). There were 18 male patients in the elderly group (13.03% [18/58]) and 56 in the very elderly group (60.22% [56/93]). The proportion of male patients in the very elderly group was significantly higher compared with that in the elderly group (*P* < 0.01).

Before treatment, there were eight patients (13.79% [8/58]) with atrial fibrillation in the elderly group and 27 patients (29.03% [27/93]) with atrial fibrillation in the very elderly group (*P* = 0.875).

The incidence of vascular lesions in the elderly and very elderly groups, respectively, was as follows: ICA (22.4% vs. 26.9%), middle cerebral artery (36.2% vs. 33.3%), BA (29.3% vs. 29%), anterior circulation (40.9% vs. 59.1%), and posterior circulation (34.9% vs. 65.1%). No significant differences were observed between the two groups (*P* > 0.05; Table 1). There was no significant difference in the baseline NIHSS score (*P* = 0.163) or the baseline mRS score (*P* = 0.935) between the elderly group and the very elderly group (Table 1). The specific infarction sites included the basal ganglia, internal capsule, cerebral lobe, watershed region, brainstem, cerebellum, and thalamus (subject to imaging). No significant difference in the number of patients with infarction in these different regions was observed between the two groups (*P* = 0.980) (Table 1).

### 3.2. Treatment Results

#### 3.2.1. mRS Score

**Figure 2:**
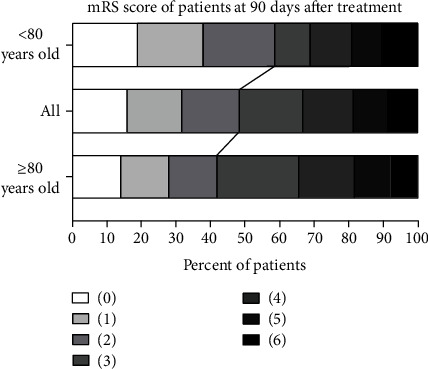
Distribution of mRS scores in the elderly group, the very elderly group, and all patients 90 days after treatment. The proportion of patients in each group with different scores was shown (0-6, the higher the score, the more severe the disability). The numbers under the bar represent the proportions of patients in different scoring segments. Due to rounding, the sum of these percentages may not equal 100. The black lines indicate the comparison of the proportion of patients with favorable outcomes (mRS 0-2 points) in different groups. The detailed results were as follows: 90 days after treatment, a total of 73 patients (48.3%) had a favorable outcome (mRS 0-2). In the group of <80 years old and ≥80 years old, 34 patients (58.6%) and 39 patients (41.6%) had a favorable outcomes, respectively. The ratio of favorable outcomes in the former was higher than that in the latter (*P* = 0.046).

Among all patients in the elderly group, 73 (48.3%) had a favorable 90-day outcome (mRS score 0–2 points). Compared with the elderly group, the very elderly group had a lower proportion of patients with a favorable outcome (41.6% vs. 58.6%, *P* = 0.046) (Figure 2**)** (Table 2).

#### 3.2.2. Death and Complications

There were 13 deaths (8.6% [13/151]) in the total cohort, and 22 patients (14.6% [22/151]) had SICH. No significant difference in the incidence of mortality or SICH was found between the elderly and the very elderly group (mortality: 10.3% vs. 7.5%, *P* = 0.548; SICH: 12.07% vs. 16.13%, *P* = 0.561) (Table 2). The differences in other complications, including respiratory failure and lung infection, were not statistically significant between the two groups (*P* = 0.9) (Table 2).

#### 3.2.3. Predictor Analysis

Table 3 shows the results of the univariate and multivariate analyses of prognostic factors at 90 days. With the univariate analysis, the factors that predicted a favorable outcome were the male sex (odds ratio (OR) = 3.243, 95% confidence interval (CI): 1.517–6.93, *P* = 0.002) and low-dose rt-PA (OR = 3.170, 95% CI: 1.373–7.320, *P* = 0.007). The factors that significantly predicted a poor outcome were sex, atrial fibrillation (OR = 4.656, 95% CI: 1.772–12.237, *P* = 0.002), and thrombolytic therapy (OR = 2.561, CI: 1.014–6.465, *P* = 0.047). The multivariate regression analysis of predictive factors indicated that the male sex and low-dose rt-PA were predictive factors for a favorable outcome.

### 3.3. Follow-Up Outcome

The duration of clinical follow-up was 90 days. The proportion of patients with a favorable outcome at 90 days (48.3% [73/151]) was significantly higher compared with that before treatment (36.4% [55/151]) (*P* = 0.036). In the elderly group, the proportion of patients with a favorable outcome at 90 days after treatment was significantly higher compared with that before treatment (58.6% vs. 31%, respectively; *P* = 0.015). The rate of a favorable outcomes at 90 days (25.8%) was not significantly different compared with the rate before treatment (24.5%) in the very elderly group (*P* = 0.791). During the follow-up period, there were five patients (3.3%) with recurrent ischemic stroke, including two patients in the elderly group and three patients in the very elderly group (3.4% vs. 3.2%, respectively; *P* = 0.941). Four patients (2.6%) developed in-stent restenosis 2 months after surgery, including one patient in the elderly group and three patients in the very elderly group (1.7% vs. 3.2%, respectively; *P* = 0.576).

## 4. Discussion

### 4.1. Effectiveness Analysis

In this study, the proportion of patients with a favorable outcome 90 days after recanalization treatment differed between the two groups; that is, the proportion of patients with a favorable outcome in the very elderly group was lower compared with that in the elderly group (Figure 2). A meta-analysis involving 1,400 subjects validated this view and proposed that the effectiveness of aortic recanalization therapy in very elderly patients was poorer compared with elderly patients [10, 14]. Most single-center trials and meta-analyses hold the view that 80 years of age is an independent predictor of functional independence and mortality 30 days after ET, and it is also the demarcation point for a rapid decline in functional prognosis [14–16]. In very elderly patients, functional improvement after treatment is limited by neuronal reserve, remodeling ability, and plasticity [14]. Another possible reason is that difficulty in vascular access and increased vascular distortion or atherosclerotic lesions may affect surgical outcomes [15, 16].

Conflicting results have been reported in previous studies. For example, Taichiro et al. showed no significant difference in the favorable outcome rate between the elderly group and the very elderly group (42% vs. 57%, respectively; *P* = 0.261), and the therapeutic effect of ET treatment between the two groups was also comparable [17]. Yasuhiro et al. proposed that age should not be used as a criterion to exclude elderly patients. They believe that improving recanalization techniques could elevate the success rate of treatment in older patients, but there is still no uniform standard on how to improve the technology [18].

In this study, we suggest that in addition to the above reasons, other baseline factors, such as atrial fibrillation and thrombolysis dose, may also be responsible for the poor prognosis of elderly patients. The lower functional independence of patients in the very elderly group before stroke may also reduce treatment effectiveness [19]. Therefore, age may indeed be a factor to consider when deciding whether recanalization therapy should be selected in the clinic.

### 4.2. Safety Analysis

In this study, we found no significant difference in safety between the two groups within 30 days after treatment. No significant difference was detected in the rate of SICH or mortality between the elderly and very elderly groups (16.1% vs. 12.1%, respectively; *P* = 0.561; 7.5% vs. 10.3%, respectively; *P* = 0.548). This may be due to the following three reasons. First, a low dose of rt-PA (0.6 mg/kg) was administered to some patients to improve safety in this study. The conditions suitable for low-dose thrombolysis were as follows: advanced age, mild stroke, relative contraindications, bleeding risk, awakening stroke, and overweight [20]. Twelve patients in the elderly group and 34 patients in the very elderly group underwent low-dose rt-PA treatment. The proportion of patients in the very elderly group who underwent low-dose rt-PA treatment was significantly higher compared with the proportion of patients in the elderly group (36.6% vs. 20.7%, respectively; *P* = 0.039). Second, careful treatment selection and active prevention of complications after treatment were adopted to further ensure patient safety. When selecting treatment for patients with a baseline mRS score of ≥2 and an ASPECT score of ≤6, a comprehensive assessment and in-depth communication with family members were carried out to ensure patient safety and successful treatment plan implementation. Finally, the interval from disease onset to treatment was strictly limited to between 4.5 and 6 hours for all patients included in the study [10]. The International Stroke Trial 3 showed no difference in the rate of cerebral hemorrhage and mortality between the very elderly group and the elderly group (4% vs. 3%, respectively; *P* = 0.667), confirming that thrombolytic therapy is safe in the elderly [21]. The results of the EXTEND-IA and REVASCAT studies have confirmed the safety of IVT and bridge endovascular therapy within 6 hours of AIS onset [22]. Therefore, our results are consistent with previous findings that the safety of recanalization treatment is comparable between the elderly and very elderly groups.

### 4.3. Influencing Factors

In this study, univariate and multivariate analyses indicated that the male sex and low-dose rt-PA thrombolysis were predictive factors for a favorable outcome. Atrial fibrillation and thrombolytic therapy were predictive of a poor outcome. According to previous meta-analyses and multicenter studies, the main factors affecting the effectiveness of recanalization treatment are baseline functional level (a higher baseline NIHSS score), vascular disease location, thrombolysis dose, time from onset to emergency, operation time, and the proportion of patients with mTICI > 2b/3 [10, 14, 23].

We found no significant difference in the baseline functional level or in the distribution of anterior-circulation and posterior-circulation lesions between the two groups in the present study (Table 1). Moreover, we found that the proportion of male patients with a favorable outcome and a poor outcome was 64.4% and 34.6%, respectively (OR = 3.24, *P* = 0.002). Findings from South Korea have shown that the average life expectancy of men is lower than that of women, resulting in a significantly decreased proportion of male patients in the very elderly group, which may have affected the results. Therefore, we speculate that the male sex may be predictive of a favorable outcome. Moreover, an older age and the female sex are also predictors of a poor outcome. Because women have a longer life expectancy than men, women are more likely to be of a higher chronological age when they experience stroke and they may thus have a higher disability rate when classified into the very elderly group [24, 25], which is consistent with previous meta-analysis results [14].

The rate of atrial fibrillation in the very elderly group was higher compared with that in the elderly group (29.03% vs. 13.79%, respectively; *P* = 0.031). The proportion of patients with atrial fibrillation in those with favorable and poor outcomes was 11% and 34.6%, respectively (OR = 4.656, *P* = 0.02), indicating that atrial fibrillation may be an influencing factor of a poor outcome. Similar findings have been reported in previous studies. The incidence of atrial fibrillation confirmed on admission was significantly higher in patients ≥ 80 years of age compared with patients < 80 years of age (69% vs. 45%, respectively; *P* = 0.042). Moreover, the incidence of cardiogenic thromboembolism in patients aged ≥80 years was significantly higher compared with those aged <80 years (94% vs. 73%, respectively; *P* = 0.016). Therefore, we infer that atrial fibrillation may lead to a higher bleeding risk and a poor prognosis. This may be due to large-artery embolism caused by atrial fibrillation, which causes infarction in the area supplied by the middle cerebral artery. Furthermore, atrial fibrillation is often associated with other high-risk factors, such as hypertension and atherosclerosis, which lead to a significantly higher mortality rate compared with patients without atrial fibrillation or patients with tremor [20, 25].

In this study, 111 patients underwent IVT and 40 patients did not undergo IVT, and there was statistical difference between the two (*P* < 0.05). A significant difference was observed between the number of patients with a poor outcome at 90 days and the number of patients with a favorable outcome (68 vs. 43, respectively; *P* < 0.001). The multivariate analysis showed that IVT is predictive of a poor outcome (*P* = 0.047), which is contrary to previous studies [15, 16, 26, 27]. With a detailed analysis of previous studies, we found that the effect of different rt-PA doses on the results was rarely mentioned. In this study, by comparing the effects of different rt-PA doses, we infer that IVT can affect the outcome of recanalization therapy, but the effect is also influenced by the rt-PA dose. In our study, the number of patients in the elderly and very elderly groups who underwent low-dose rt-PA treatment (0.6 mg/kg) was significantly different (13 [20.6%] vs. 50 [79.4%], respectively; *P* = 0.001). Therefore, we speculate that low-dose rt-PA thrombolysis may increase safety but significantly reduce the effectiveness of treatment, thus making IVT a predictive factor for a poor outcome in this study. Similar to our results, Dong et al. recently reported that low-dose rt-PA thrombolysis can reduce the risk of SICH, but it also significantly reduces treatment effectiveness [28].

In previous studies, the influence of age and serum 25 (OH) D concentration on the prognosis of stroke has been noted. According to the DNA methylation algorithm, biological age is probably an independent predictor of 3-month mortality after ischemic stroke, which is unrelated to NIHSS score and vascular risk factors [29]. Tu et al. suggest that 25 (OH) D remains an independent prognostic marker of 90-day mortality and functional outcomes in Chinese patients with AIS; lower serum levels of 25 (OH) D are independently associated with the stroke recurrence and mortality at 24 months in ischemic stroke patients [30, 31]. Cheng et al. suggested that elevated serum hS-CRP and HCY levels were associated with the risk of PSD 1 year after stroke, and this was likely to be a factor influencing the favorable outcomes at 90 days [32]. Further randomized controlled trials are needed to assess the factors that influence the prognosis of stroke in elderly patients.

This study had several limitations. First, this study was a single-center study; thus, the evaluation may not be sufficiently comprehensive. There is a need to further expand the scope of analysis to include other factors, such as ASPECT score, collateral circulation grading, and the relatively small number of cases after grouping. Second, the overall sample size was small, and this limitation was particularly prominent when the three methods were separately counted. This may have led to a decrease in detection efficiency. In future studies, the inclusion criteria should be optimized, the number of subjects should be increased, and data from multiple research centers should be combined for analysis.

## 5. Conclusion

In this study, nearly half of the total cohort achieved a good functional prognosis at 3 months after recanalization. Recanalization had comparable safety between patients aged ≥80 years and patients aged 60–79 years. Although there were no significant differences between the two groups in terms of group size, baseline functional score, and vascular lesions, the benefits of recanalization in very elderly patients were not as good as those in elderly patients. Therefore, we infer that age may be an important factor affecting the outcome of aortic recanalization. Furthermore, we identified other influencing factors. Specifically, sex may affect treatment effectiveness, low-dose rt-PA thrombolysis may improve treatment safety, and atrial fibrillation may predict a poor prognosis. In clinical practice, a comprehensive evaluation of certain factors, such as age, must be considered to achieve better treatment outcomes.

## Figures and Tables

**Table 1:** Comparison of baseline characteristics between <80 and ≥80 groups.

Clinical data		All (*n* = 151)	<80-years-old group (*n* = 58)	≥80-years-old group (*n* = 93)	Test used	*x* ^2^/*t*/*Z*	*P*
Gender	Male (*n* (%))	74 (49.01)	18 (31.03)	56 (60.22)	Chi-square test	12.172^a^	<0.001
	Female (*n* (%))	77 (50.99)	40 (68.97)	37 (39.78)			
BMI		24.66 ± 1.73	24.47 ± 1.83	24.77 ± 1.67	*t*-test	-1.036^b^	0.302
Time from onset to groin puncture (hours)		6.29 ± 0.72	6.26 ± 0.68	6.30 ± 0.76	*t*-test	-0.349^b^	0.727
Time from onset to recanalization (min)		40.43 ± 2.98	40.24 ± 3.16	40.55 ± 2.87	*t*-test	-0.615^b^	0.539

Baseline medical history	Hypertension (*n* (%))	59 (39.07)	23 (39.66)	36 (38.71)	Chi-square test	0.013^a^	0.908
	Diabetes (*n* (%))	55 (36.42)	21 (36.21)	34 (36.56)			
	Hyperlipidemia (*n* (%))	48 (31.79)	18 (31.03)	30 (32.26)			
	Coronary heart disease (*n* (%))	53 (38.48)	21 (29.97)	32 (34.41)	Chi-square test	0.002^a^	0.965
	Atrial fibrillation (*n* (%))	35 (23.18)	8 (13.79)	27 (29.03)	Chi-square test	0.025^a^	0.875

Vessel involved	Middle cerebral artery (*n* (%))	43 (28.48)	21 (36.2)	31 (33.3)	Chi-square test	0.418^a^	0.741
	Internal carotid artery (*n* (%))	31 (20.53)	13 (22.4)	25 (26.9)			
	Basilar artery (*n* (%))	35 (23.18)	17 (29.3)	27 (29)			
	Vertebral artery (*n* (%))	13 (8.61)	7 (12.1)	10 (10.8)			
	Anterior cerebral artery	88 (58.3)	36 (40.9)	52 (59.1)	Chi-square test	0.557^a^	0.456
	Posterior cerebral artery	63 (41.7)	22 (34.9)	41 (65.1)			

Occlusion site	Basal ganglia	33 (21.9)	13 (24.5)	20 (20.4)	Chi-square test	1.312	0.980
	Inner capsule	7 (4.6)	2 (3.8)	5 (5.1)			
	Brain lobe	26 (17.2)	10 (18.9)	16 (16.3)			
	Watershed	22 (14.6)	7 (13.2)	15 (15.3)			
	Brain stem	41 (27.2)	13 (24.5)	28 (28.6)			
	Cerebellum	10 (6.6)	3 (5.7)	7 (7.1)			
	Thalamus	12 (7.9)	5 (9.4)	7 (7.1)			

Baseline NIHSS, median (IQR)		9 (4, 16)	11 (6, 15.25)	8 (3, 16)	Wilcoxon rank sum test	4.659^a^	0.163
Baseline mRS, median (IQR)		3 (2, 4)	3 (2, 4)	3 (2, 4)	Wilcoxon rank sum test	0.425^a^	0.935
Acute treatment (rt-PA)					Chi-square test	4.247^a^	0.001
0.6 mg/kg		63 (56.8)	13 (20.6)	50 (79.4)			
0.9 mg/kg		48 (43.2)	10 (20.8)	38 (79.2)			

a means chi-square test; b stands for *t*-test.

**Table 2:** Comparison of treatment outcomes between the elderly group and the very elderly group.

	All	<80 (58)	≥80 (93)	*P*
mRS at 90 days (*M*, IQR)	3 (1, 4)	2 (1, 4)	3 (1, 4)	0.246
IVT (*M*, IQR)	3 (1, 4)	2 (1, 4.5)	3 (1, 4)	0.450
ET (*M*, IQR)	3 (2, 4)	3 (1.75, 5)	2 (0, 4)	0.486
IVT&ET (*M*, IQR)	2 (1, 4)	2 (0, 4)	3 (1, 4)	0.173
Before treatment Favorable outcomes (mRS 0-2 at 90 days) (*n*, %)	55 (36.4)	18 (31.0)	37 (39.8)	0.277
90 days after treatment Favorable outcomes (mRS 0-2 at 90 days) (*n*, %)	73 (48.3)	34 (58.6)	39 (41.6)	0.046
Other outcome (mRS 3-6 at 90 days) (*n*, %)	78 (51.7)	24 (41.4)	54 (58.1)	0.046
SICH (*n*, %)	22 (14.6)	7 (12.07)	15 (16.13)	0.561
Other complications (*n*, %)	46 (30.5)	18 (31.0)	28 (30.1)	0.904
Death (mRS 6 at 90 days) (*n*, %)	13 (8.6)	6 (10.3)	7 (7.5)	0.548
mRS at 90 days in different degrees (*n*, %)				0.380
0	24 (15.9)	11 (19.0)	13 (14.0)	
1	24 (15.9)	11 (19.0)	13 (14.0)	
2	25 (16.6)	12 (20.7)	13 (14.0)	
3	28 (18.5)	6 (10.3)	22 (23.7)	
4	22 (14.6)	7 (12.1)	15 (16.1)	
5	15 (9.9)	5 (8.6)	10 (10.8)	
6	13 (8.6)	6 (10.3)	7 (7.5)	

Normally distributed data is presented as mean ± standard deviation and compared using independent sample *t*-test. Data that are not normally distributed is expressed as *M* (P25-P75), and the Wilcoxon rank sum test is used for the comparison between groups. Count data is represented by *n* (%), and the comparison between groups is done by the chi-square (*χ*^2^) test. The multivariate analysis is tested by two-class logistic regression analysis. *P* < 0.05 indicates statistical significance.

**Table 3:** Multivariate analysis for predictors of good outcome and poor outcomes.

	Univariate logistic regressions for outcome			Multivariate logistic regressions for outcome	
	Favorable outcomes *n* = 73	Poor outcomes *n* = 78	*P*	Odds ratio (95%)	*P*
Male	47 (64.4)	27 (34.6)	<0.001^a^	3.243 (1.517, 6.93)	0.002
BMI	24.71 ± 1.68	24.61 ± 1.79	0.726		
Onset to groin time (h)	6 (6, 7)	6 (6, 7)	0.684^b^		
Onset to IVT time (min)	40 (38, 43)	40.5 (38, 43)	0.419^c^		
Baseline NIHSS	9 (4, 15)	9 (4, 16)	0.761^c^		
Hypertension	24 (32.9)	35 (44.9)	0.131^a^		
Diabetes mellitus	31 (42.5)	24 (30.8)	0.136^a^		
Hyperlipidemia	19 (26)	29 (37.2)	0.141^a^		
Coronary heart disease	21 (35.1)	32 (41)	0.143^a^		
Arterial fibrillation	8 (11)	27 (34.6)	0.001^a^	4.656 (1.772, 12.237)	0.002
Location			0.565^a^		
Middle cerebral artery (*n* (%))	24 (32.9)	28 (35.9)			
Internal carotid artery (*n* (%))	21 (28.8)	17 (21.8)			
Basilar artery (*n* (%))	22 (30.1)	22 (28.2)			
Vertebral artery (*n* (%))	6 (8.2)	11 (14.1)			
Treatment options			0.271^a^		
Age			0.046^a^	3.536 (1.344, 9.306)	0.011
<80	34 (46.6)	24 (30.8)			
≥80	39 (53.4)	54 (69.2)			
rt-PA			<0.001^a^	2.561 (1.014, 6.465)	0.047
No	30 (41.1)	10 (12.8)			
Yes	43 (58.9)	68 (87.2)			
rt-PA 0.6 mg/kg	32 (43.8)	14 (17.9)	0.001^a^	3.170 (1.373, 7.320)	0.007

“a” indicates chi-square test; “b” indicated *t*-test; “c” indicates *Z* test. The assignment is as follows: favorable outcomes group = 1, poor prognosis group = 0; 0.6 mg/kg = 1, and 0.9 mg/kg = 2. BMI: body mass index.

## Abbreviations

NIHSS: The National Institutes of Health Stroke Scale
mRS: The modified Rankin scale
DSA: Digital subtraction angiography
AIS: Acute ischemic stroke
LAO: Large artery occlusion
SICH: Symptomatic intracranial hemorrhage
ET: Endovascular thrombectomy
IVT: Intravenous alteplase
rt-PA: Recombinant tissue plasminogen activator
ASPECT: Alberta Stroke Program Early CT
DWI: Diffusion-weighted magnetic resonance imaging
CT: Computed tomography
ICA: Internal carotid artery
VA: Vertebral artery
BA: Basilar artery
M1: Main middle cerebral artery
MRA: Magnetic resonance angiography
mTICI: Modified thrombolysis in cerebral infarction.
